# Potential role of miRNA-140 in Alzheimer’s disease

**DOI:** 10.18632/aging.101827

**Published:** 2019-02-19

**Authors:** Rumana Akhter, Lynn M. Bekris

**Affiliations:** 1 Cleveland Clinic Lerner Research Institute, Cleveland, OH 44106, USA

**Keywords:** Alzheimer’s disease, ADAM10, micro-RNA, β-amyloid

Alzheimer's disease (AD) is the most common cause of dementia and one of the leading causes of death in aging population worldwide. It is estimated that more than 50 million people around the globe are affected with AD. The accumulation of toxic amyloid plaques, hyperphosphorylated tau tangles and extensive neuronal degeneration are hallmark features of AD [1]. MicroRNA (miRNA) expression levels have been shown to change dramatically in response to various cell stressors and in neurodegenerative disease [2]. MiRNAs are small non-coding RNAs approximately 20–24 nucleotides long with important biological activities, primarily related to post-transcriptional regulation of gene expression. These small RNA moieties are conserved throughout evolution, and their expression can be constitutive and tightly regulated. Subsets of miRNA are specifically expressed in certain regions of the brain or in neuronal and glial cell subtypes as a means to assure regional or cell type gene regulation [3]. The studies of microRNAs expression profiles in nervous system represent important steps in understanding the role of miRNAs in regulating gene expression. Increasingly research efforts are focusing on identifying specific miRNA as potential therapeutic targets for the regulation of candidate disease genes. Multiple reports suggest that alterations in miRNA expression in human AD brain are associated with disease pathology [4].

In AD, the principal neurotoxic factor β-amyloid is formed through the sequential cleavage by β- and γ-secretase enzymes of the amyloid precursor protein (APP) [1]. An alternative nonamyloidogenic pathway exists where APP is cleaved by A Disintegrin and Metalloproteinase 10 (ADAM10). ADAM10 is a critical physiological α-secretase present in brain with beneficial neurotrophic functions through both the inhibition neurotoxic Aβ production and in turn the production of the soluble amyloid precursor protein (sAPPa), which has neuroprotective properties [5]. ADAM10 is decreased in AD brain, but little is known about the underlying mechanisms involved in this decrease. In our recent study we have elucidated the role of miRNA-140-5p in regulation of ADAM10. Two types of gene regulatory factors; transcription factors and miRNA can greatly influence gene expression. Transcription factors regulate transcription either positively or negatively whereas miRNAs regulate gene expression mostly through repression [6]. Among the pool of our studied miRNA, miRNA-140-5p was found to be upregulated in AD hippocampus compared to controls. This miRNA-140-5p was also found to be elevated in neuronal SHSY5Y cells and associated with a significant decrease in ADAM10 reporter construct activity under Aβ toxicity. Interestingly, others have described an upregulation of miR-140-5p in blood from patients with acute ischemic stroke suggesting miR-140 production may increase in response to neurological injury [7]. Furthermore, reportedly miR-140-5p expression in hippocampal CA1 neurons from prion infected mice significantly correlates with genes involved in neuronal projection and dendrite development implicating a critical role by miR-140-5p in neuronal processes [8].

Another important aspect of our study was to determine if miRNA-140-5p plays a broader role in the regulation of ADAM10, such as the regulation of ADAM10 transcription factors. Transcription factor candidates were selected based on the following criteria; whether miR-140-5p was predicted to target the transcription factor gene, there was previous evidence of transcription factor activity in neurodegenerative disorders, and whether it had been previously described as a regulator of ADAM10. SRY (sex determining region Y)-box 2 also known as SOX2 fit these criteria. Therefore, next we focused on elucidating the functional relationship between the trans-acting factors SOX2 and miRNA-140-5p in ADAM10 regulation. Overexpression of SOX2 increased ADAM10 reporter construct activity indicating that SOX2 directly regulates ADAM10 promoter activity. In addition, site directed mutagenesis creation of a mutation in the miR-140 binding site, in both the ADAM10 3’UTR and the SOX2 3’UTR, ablated miR-140-5p inhibition of SOX2 and ADAM10 reporter activity. These findings suggest that miR-140-5p inhibits the production of ADAM10 both through regulation of the SOX2 transcription factor and through direct regulation of ADAM10. This information may also have wider ramifications for other APP cleavage products since ADAM10 cleavage also produces the neuroprotective soluble APP-α. Therefore, therapeutic inhibition of miR-140-5p *in vivo* to increase both SOX2 and ADAM10 levels may offer a feasible approach for AD intervention. This approach will have the advantage of both enriching the level of soluble APP-α and reducing Aβ levels. Our findings highlight the potential of combinatorial regulation of transcription factors and microRNAs in the targeted regulation of ADAM10 and provides a strong basis for future research aimed at annotating trans-acting regulatory factors as therapeutic targets in AD.

## References

[1] Hardy J, Selkoe DJ. Science. 2002; 297:353–56. 10.1126/science.1072994 12130773

[2] Nelson PT, et al. Brain Pathol. 2008; 18:130–38. 10.1111/j.1750-3639.2007.00120.x 18226108 PMC2859437

[3] Kosik KS. Nat Rev Neurosci. 2006; 7:911–20. 10.1038/nrn2037 17115073

[4] Burgos K, et al. PLoS One. 2014; 9:e94839. 10.1371/journal.pone.0094839 24797360 PMC4010405

[5] Fahrenholz F, et al. Ann N Y Acad Sci. 2000; 920:215–22. 10.1111/j.1749-6632.2000.tb06925.x 11193153

[6] Hobert O. Science. 2008; 319:1785–86. 10.1126/science.1151651 18369135

[7] Sørensen SS, et al. Transl Stroke Res. 2014; 5:711–18. 10.1007/s12975-014-0364-8 25127724

[8] Majer A, et al. PLoS Pathog. 2012; 8:e1003002. 10.1371/journal.ppat.1003002 23144617 PMC3493483

